# Return to work and clinical outcome after surgical treatment and conservative management of patients with intramedullary spinal cord ependymoma

**DOI:** 10.1038/s41598-020-59328-1

**Published:** 2020-02-11

**Authors:** Bedjan Behmanesh, Florian Gessler, Sae-Yeon Won, Daniel Dubinski, Johanna Quick-Weller, Lioba Imoehl, Volker Seifert, Gerhard Marquardt

**Affiliations:** Department of Neurosurgery, Goethe- University, Frankfurt am Main, Germany

**Keywords:** Spinal cord diseases, Surgical oncology

## Abstract

The ability to return to work after treatment of diseases is an important issue. Aim of this study is to compare surgery and conservative management focusing on clinical outcome and ability to return to work in patients with intramedullary spinal cord ependymoma. Retrospective, single center study. The neurological status at first presentation, as well as in long-term follow-up, were assessed using the modified McCormick Disability Scale and modified Rankin Scale. The study population consisted of 56 patients, 23 (41%) were managed conservatively and 33 (59%) underwent microsurgical resection. The median age was 47.5 years in the conservative group and 44.5 in the surgical group. At first admission 18 of conservatively treated and 28 of surgically treated patients were employed, *p* = 0.7. At the last follow-up 15 (83%) of conservatively and 10 (36%) of surgically treated patients returned to work, *p* = 0.002. The median modified McCormick score in both groups (conservative vs. surgical) was at admission 1 vs. 1, *p* = 1.0 and at last follow up 1 vs. 2.5, *p* = 0.001. Patients clinical outcome in the surgical group was significantly reduced at last follow up as assessed by the modified Rankin Scale (mRs score of 0–2) at admission 100% vs. 100% and last follow-up 94% vs. 57%, *p* = 0.007. In our investigated study population, conservatively managed patients revealed a significantly better outcome and were more often able to return to work.

## Introduction

According to the results of a survey from 2015 among the German population in the age between 18 and 60 years, work and being employed ranged as an important factor of quality of life after family and partnership^1^. Being employed is synonymous with a normal life and is regarded as a marker of complete recovery after treatment of several diseases. Moreover, employment provides a sense of structure, income and identity^2,3^. Until now many data have been published analyzing and assessing treatment approaches and the effect of surgery on patient’s outcome, but few data exist evaluating patients’ outcome after conservative management^4–8^. To the best of our knowledge no data have been published with focus on work ability and return to work after treatment, either surgical or conservative management of patients with intramedullary spinal cord ependymoma.

## Results

The study cohort included 56 patients with a median age of 51 ± 13.8 years. The gender distribution included 24 (42.9%) females and 32 (57.1%) males. Subsequently 33 (58.9%) patients underwent surgery and 23 (41.1%) preferred conservative management with follow-up examination and MRI. At first admission 28 (84.8%) from 33 patients with recommendation for surgery and 18 (78.3%) from 23 patients from the conservatively treated group were in working age and were then included in this study. Table 1 entails further patients data.

**Table 1:** Patients characteristics of conservatively and surgically treated patients.

Characteristics	conservatively managed	surgically treated	P value
No. patients	23	33	
**No**. **patients in working age**	**18 (78**.**3%)**	**28 (84**.**8%)**	0.7
Age (mean)	47.5 ± 10.5 years	44.5 ± 10.5 years	0.3
**Sex**			
Male	9 (%)	18	0.4
Female	9 (%)	10	
**Follow Up (mean/range)**	53.5 (4–118) months	81.8 (3–266) months	0.4
**Location**			
Cervical	12	13	0.2
Thoracic	6	12	0.6
Cervicothoracic		2	
Conus		1	
**Involved levels**			
1	14	20	0.7
>1	4	8	0.7
**Initial symptoms**			
Pain	2	5	0.7
Paresthesia	11	4	0.002
Pain+ Paresthesia	0	13	0.0005
Motor weakness	1	3	1.0
No symptoms	4	1	0.07
other	0	2	0.5
Duration of symptoms average (range)	10.6 (1–48) months	11.9 (1–48) months	
Tumor resection			
total		25	
subtotal		2	
biopsy		1	
**Histology**			
WHO I		1	
WHO II		25	
WHO III		2	
**Jobs before treatment**			
Physical work	8	8	0.3
Office work	4	6	1.0
Intellectual work	6	9	1.0
House-maker		4	0.1
incapacitated		1	1.0

### Surgical group

Twenty eight of 33 subsequently surgically treated patients were employed at first admission. The median age in this group was 44.5 ± 10.5 years with a gender distribution of 10 (35.7%) females and 18 (64.3%) males. The tumor was located in the cervical spine in 13 (46.4%) patients, in the thoracic spine in 12 (42.9%) patients, in the cervicothoracic region in two (7.1%) patients and in the conus medullaris in one (3.6%) patient. The intramedullary spinal cord ependymoma affected one spinal cord level in 20 and more than one level in 8 patients. The predominant symptoms, which lead to admission, were pain and paresthesia in 13 (46.4%) cases, pain without sensory deficit in five (17.9%) cases, paresthesia without further signs in four (14.3%) patients, mild motor dysfunction in three (10.7%) patients and incidental finding in one (7.1%) patient. Clonus as pathological finding was present in one patient (7.1%) and dyssynergia in another patient (7.1%). Total resection of the tumor could be achieved in 25 (89.3%) cases and subtotal resection in two cases (7.1%). Mere biopsy of the lesion was performed in only one case (3.6%).

**Figure 1:**
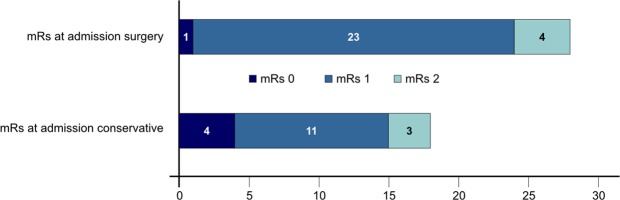
Modified Rankin Scale (mRs) at admission in surgically and conservatively managed patients at admission.

Histological workup after surgery revealed WHO I ependymoma in only one (3.6%) patient, WHO III in two (7.1%) patients and WHO II in 25 (89.3%) cases. The median modified McCormick score in surgically treated patients at admission was 1 ± 0.38 (SD) and according to the modified Rankin Scale all patients were in good functional condition (mRs 0–2), which indicates functionally independency, Fig. 1.

Compared to initial neurological status 9 patients remained unchanged postoperatively, of which three patients improved and one patient deteriorated at the last follow-up. Postoperative neurological decline was seen in 19 patients, of which 6 patients improved and 5 patients remained unchanged in further clinical course.

**Figure 2:**
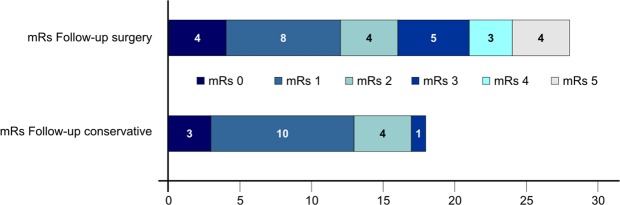
Modified Rankin Scale (mRs) at admission in surgically and conservatively managed patients in follow-up.

After a median follow-up time of 81.8 months the median McCormick score in surgically treated patients was 2.5 ± 1.4, *p* = 0.001. Functional independency (mRs 0–2) could be proved in 16 patients (57.1%) as compared to 100% initially, *p* = 0.0001 Fig. 2.

Tumor recurrence as reason for clinical deterioration could be excluded in all cases. Eight patients (28.6%) performed physical work, nine patients (32%) had intellectual work, six (21.4%) patients performed office jobs, four patients (14.3%) were housewives and one patient was incapacitated.

At the last follow-up only 10 (35.7%) patients returned to work. From further 18 patients undergoing surgical tumor resection and not returning to work after surgery, five patients had an office jobs prior to surgery, four were housemakers, intellectual work were performed in four cases and physical work in four cases. One patient could not work prior to surgery in order to incapacity and remained unemployment, Table 2.

**Table 2:** Functional outcome at admission and at last follow up.

	Conservatively managed	Surgically treated	P value
mMcC at admission (median)	1	1	1.0
mMcC at last follow up (median)	1	2.5	0.001
mRS 0–2 at admission	100%	100%	1.0
mRS 0–2 at last follow up	94%	57%	0.007
Return to work	15	10	0.002

### Conservative group

At the time of first of introduction to our department 18 from 23 patients were employed. The median age in this group was 47.5 ± 10.5 years. There was an equal gender distribution consisting of nine females and nine males. The most affected spinal cord region in this group was the cervical spine in 12 (66.7%) cases, followed by the thoracic region in six (33.3%) cases. The tumor involved one level of the spinal cord in 14 (77.7%) patients, two levels in three (16.7%) cases and three levels in one (5.6%). At first presentation only one patient revealed moderate motor dysfunction, whereas the main leading symptoms were dysesthesia and pain. Incidental finding as result of headache workup was the reason for initial introduction in four (22.2%) patients, Table 3.

**Table 3:** Demographics and clinical outcomes of 18 patients undergoing conservative management.

Patients	Age (years)	Sex	Duration of symptoms (months)	mMcC at admission	mMcC at last FU	mRS at admission	mRS at last FU	Radiological progression
1	47	F	4	2	2	2	2	
2	55	M	4	1	3	1	3	Yes
3	34	F	3	1	1	1	1	
4	54	F	48	1	1	1	1	
5	58	F	5	2	2	1	1	
6	62	M	12	1	2	1	2	No
7	50	M	2.5	1	1	1	1	
8	53	F	1	2	2	2	2	
9	51	F		1	1	0	0	
10	46	M	48	2	2	2	2	
11	28	M	6	1	2	1	1	No
12	51	F		1	2	0	1	
13	38	F		1	1	0	0	
14	26	M		1	1	0	0	
15	55	F	1	1	1	1	1	
16	59	M	8	1	1	1	1	
17	48	M	3	1	1	1	1	
18	40	M	3	1	1	1	1	

FU: follow-up.

The median modified McCormick score at presentation in these patients was 1 ± 0.4 (SD) and the modified Rankin Scale consisting of 0–2 was 100%, Fig. 1. Over time a decline in the neurological function was found in three (16.7%) patients, whereas only one patient revealed severe ataxia. Two of the three patients underwent surgery. Tumor progression detected on follow-up MRI was seen in one patient undergoing surgery, whereas the other one revealed a clinical deterioration, in the form of dysesthesia. The clinical decline in the third patient was little, mild increase of paresthesia, and did not justify surgical intervention. The median modified McCormick score after a median follow-up period of 53.5 months was 1.0 ± 0.6, *p* = 0.1. The modified Rankin Scale in one patient was 3 and in all other patients remained between 0–2 (94.4%), *p* = 1 Fig. 2.

From 18 patients 15 patients (83.3%) remained employed at the last follow up. The reason for unemployment was neurological detoriation in one patient and two patients (11.1%) did not feel like working again and intended to apply for disability pension. Eight patients (44.4%) performed physical work, six patients (33.3%) had intellectual work, four (22.2%) patients performed office jobs and five patients (27.7%) were retired. All three patients, who did not return to work performed physical work.

### Intergroup comparison

No significant differences were obtained analyzing number of investigated patients, mean age, gender distribution, length of follow-up, location and number of involved levels affected by the tumor. The different professional activities were almost similar in both groups, as well. Paresthesia was more often proven in the conservative group (11 vs. 4, p = 0.002) and pain in combination with paresthesia in the surgical group (0 vs. 13, p = 0.005).

The median modified McCormick score between surgically and conservatively treated patients at admission did not differ significantly and was 1 ± 0.4 and 1 ± 0.38 in both groups, respectively, *p = *1.0. At last follow-up there was a decline in neurological function in surgically treated patients as assessed by the mMcC (1.0 ± 0.6 vs. 2.5 ± 1.4, *p* = 0.001), Table 2. The level of independency and good neurological function was assessed using the modified Rankin Scale and was defined between 0–2. At first admission 100% of conservatively and surgically treated patients were ranked between mRs 0–2. At last follow-up there was a decline in neurological function in both groups. 94% of conservatively managed patients revealed a neurological status according to mRs 0–2, whereas only 57% of surgically treated patients were functionally independent and ranked between mRs 0–2 (*p* = 0.007 OR 12.75, CI 1.5–109.6).

At first admission 18 patients of conservatively managed patients and 28 patients of surgically treated patients were employed, *p = *0.7. At last follow-up after a median follow-up time of 81.8 and 53.5 months, respectively, 15 (83%) of conservatively and 10 (36%) of surgically treated patients returned to work, p = 0.002 OR 9, CI 2.1–38.8, Table 2.

Focusing on patients with a long follow-up period ( > 4 years) there were 19 patients in the surgical arm and 9 patients in the conservative arm. The median initial neurological status according to the mMcC were 2 ± 0.5 and 1 ± 0.4, respectively and did not differ significantly, *p* = 0.1. All patients in both arms were employed at initial presentation. At the last follow-up the mMcC in both groups were 2 ± 1.4 and 1 ± 0.7, *p* = 0.01 and 9 (47%) patients undergoing surgery vs 8 (89%) patients being treated without surgery returned to work, *p* = 0.05.

## Discussion

Intramedullary spinal cord ependymoma is a rare entity accounting for 60% of all intramedullary spinal cord neoplasms^7,9,10^. Despite increasing understanding of the disease, radiological imaging progress, wide access and availability of electrophysiological monitoring and improved microsurgical techniques the surgical removal of intramedullary ependymoma still remains challenging. Neurological deterioration after tumor removal remains a considerable disadvantage in patients. Conservative management of these patients has been described only once consisting of a single center case series^11^. In addition, until now no scientific data exist describing the ability to return to work after diagnosis and therapy of intramedullary spinal cord ependymoma. Aim of the present study is to elucidate the level of independency and moreover the work capacity after surgical and conservative treatment of intramedullary spinal cord ependymoma.

The role of work in our modern societies has become a central feature of economic and social life.

In addition, work can be seen as a source of socializing, self-confidence, physical and mental health and self-worth. Therefore, it is a crucial issue to evaluate the level of disability and work ability as indicator of successful treatment. Hence, many authors provided case series describing the functional outcome and resumption of work in many diseases, such as subarachnoid hemorrhage, glioma surgery, spine surgery, traumatic brain injury, nerve and plexus injury^12–17^.

The diagnosis of intramedullary spinal cord lesion has an enormous impact on patient’s life, level of present or upcoming disability and ability to continue to work. Despite the benign character of the lesion, the potential compression of the spinal cord leads in many cases to severe disability. In many cases surgery is advocated and also performed, but reviewing the current published data the risk of permanent disability is accounting between 3% and 50%^6,7,18–21^.

Even after complete tumor removal, good clinical condition after surgery and without proven tumor recurrence many patients reveal a decline in their neurological status induced more likely by microstructural atrophic changes within the spinal cord tissue^18^. Another issue is the lack of data concerning the functional outcome of conservatively managed patients. All together lead to the conclusion that the most appropriate therapy of intramedullary spinal cord lesions remains still challenging and that a multidisciplinary approach should be undertaken with consideration of neurological outcome and good quality of life.

Nevertheless, the mainstay of treatment of patients harboring symptomatic intramedullary spinal cord ependymoma is the microsurgical resection of the lesion. According to our institutional standard, patients with newly diagnosed symptomatic intramedullary spinal cord ependymoma were counseled for surgery. Surgery was indicated and recommended in all patients with neurological deficits, such as motor dysfunction, sensory deficit or bowl and bladder impairment. Moreover, there were two time periods how surgical intervention and conservative surveillance were recommended. In the beginning (1980–2000) surgery was recommended in all cases on initial presentation according to the pertaining view of treatment in these patients published by many authors. Conservative treatment was initially established in patients denying surgical treatment. All these patients underwent follow-up MRI and clinical examination to detect any changes in their clinical course. After having a group of patients, who primarily refused surgery, we found that these patients had a stable follow-up without progression of the disease. Thereafter, in some patients without neurologic deficit conservative management was recommended as well in accordance to our own institutional “experience”.

After 2000 radiological surveillance was recommended as well and surgery was then performed in cases of tumor progression and neurological decline.

Therefore, we were able to evaluate the clinical outcome in these patients. Based on the results discussed above and in knowledge of surgery-related morbidity in patients with intramedullary spinal cord ependymoma, surgery might not be necessarily recommended in all patients. In some cases, especially presenting with mild or no symptoms conservative management can be an alternative, as well. But, in our patient’s cohort two patients must undergo surgery in the follow up period due to neurological deterioration in order of tumor growth over time.

One aspect of good clinical outcome is the ability to return to work, which is not highlighted in this patient’s population. By comparing the basic patient’s characteristics and without identification of significant differences with regard to sex, age, affected spinal cord location of the lesion and involved spinal cord level, we could demonstrate that both groups are almost similar. We could also show that the initial neurological status at first introduction was similar, as well, which is the main aspect in case of detecting any differences affecting quality of life and work capacity over time. In our investigated patients population there was a significant difference in clinical outcome in patients undergoing surgical resection of their spinal cord lesions and that significantly more patients managed conservatively could return to work.

### Study limitation

This study is limited by its retrospective design. Due to the benign entity intramedullary spinal cord ependymoma have a slow growth tendency. Therefore, it is mandatory to assess the clinical course of patients with a long follow-up period. Although most patients in our study had a long follow-up period, there were some patients with a relatively short follow-up. A longer follow-up period with serial imaging would have been useful to get some measure of the biology and natural history of these tumors.

## Conclusion

Return to work or remaining employed is an important issue of good quality of life. The majority of patients, who underwent conservative management, revealed a stable functional status in the long term follow up and could significantly more often resume their working activity.

## Material and Methods

Retrospective, single center review of all patients with intramedullary spinal cord ependymoma treated between 1980 und 2017. The neurological outcomes pre- and postoperatively and in conservatively managed patients, as well as long-term follow-up, were assessed using the modified McCormick Disability Scale (mMcC) and modified Rankin Scale. Diagnosis in conservatively treated patients based on MRI scan that were rated by both an experienced radiologist and the senior author as being consistent with an intramedullary spinal cord ependymoma in conservatively treated patients. Surgery was performed using a standard dorsal approach with laminectomy. In selected cases with multilevel tumor involvement a laminoplasty was performed instead. The amount of tumor resection was rated as total resection, subtotal resection or biopsy. Total tumor resection was defined as 100% tumor removal, if confirmed by the surgeon and postoperative MRI scan.

Good outcome was defined as stable mMcC score or improvement in mMcC score in comparison to the condition at admission. According to the pertaining treatment recommendation surgery was advocated and performed in patients with assumed diagnosis of IMSCE in our department, as well. In cases of asymptomatic or mild symptomatic tumors and in knowledge of potential occurrence of neurological disability after surgery some patients refused surgery and advocated routinely MRI and follow-up examination. All these patients underwent further clinical and radiological follow-up. In cases of symptom progression or tumor growth over time surgery was recommended also in patients being initially treated conservatively. The primary outcome measure was the shift in the degree of disability among patients as measured by the modified Rankin Scale (mRS) (with scores ranging from 0 [fully independent] to 6 [dead]) at follow up and McCormick score (with scores ranging from 1 [neurologically intact] to 5 [paraplegia, quadriplegia], as well. Routinely performed electrophysiological examination was analyzed to detect neurological deterioration within the follow up period^11^.

### Statistical analysis

All statistics were performed using SPSS (version 21, IBM, Armonk, New York). Significant values were considered to be P < 0.05. Comparison of important differences between the study groups was made using Fishers exact test for categorical variables. Nonparametric tests included the Mann–Whitney U and Kruskal–Wallis test to compare groups of data that did not follow the normal distribution. Tests for normality were performed using the Shapiro–Wilk test.

### Ethical approval and informed consent

This study was approved by the local ethics committee of the Goethe University Frankfurt am Main, the methods were carried out in accordance with the relevant guidelines and regulations. Informed consent was obtained in each case.

## Data Availability

The datasets generated during and/or analysed during the current study are available from the corresponding author on reasonable request.
